# Penetration of biomass-burning emissions from South Asia through the Himalayas: new insights from atmospheric organic acids

**DOI:** 10.1038/srep09580

**Published:** 2015-04-09

**Authors:** Zhiyuan Cong, Kimitaka Kawamura, Shichang Kang, Pingqing Fu

**Affiliations:** 1 Key Laboratory of Tibetan Environment Changes and Land Surface Processes, Institute of Tibetan Plateau Research, Chinese Academy of Sciences, Beijing 100101, China; 2 Institute of Low Temperature Science, Hokkaido University, Sapporo. 060-0819, Japan; 3 CAS Center for Excellence in Tibetan Plateau Earth Sciences, Beijing 100101, China; 4 State Key Laboratory of Cryospheric Sciences, Cold and Arid Regions Environmental and Engineering Research Institute, CAS, Lanzhou 730000, China; 5 LAPC, Institute of Atmospheric Physics, CAS, Beijing 100029, China

## Abstract

High levels of carbonaceous aerosol exist over South Asia, the area adjacent to the Himalayas and Tibetan Plateau. Little is known about if they can be transported across the Himalayas, and as far inland as the Tibetan Plateau. As important constituents of aerosols, organic acids have been recognized as unique fingerprints to identify the atmospheric process. Here we measured dicarboxylic acids and related compounds in aerosols on the northern slope of Mt. Everest (Qomolangma, 4276 m a.s.l.). Strong positive correlations were observed for dicarboxylic acids with biomass burning tracers, levoglucosan and K^+^, demonstrating that this area was evidently affected by biomass burning. The seasonal variation pattern of dicarboxylic acids is consistent with OC and EC, being characterized by a pronounced maximum in the pre-monsoon season. Molecular distributions of dicarboxylic acids and related compounds (malonic acid/succinic acid, maleic acid/fumaric acid) further support this finding. We suggest that the local meteorological conditions and regional atmospheric flow process could facilitate the penetration of the carbonaceous aerosols from South Asia throughout the Himalayas. With the consideration of the darkening force of carbonaceous aerosols, our finding has important implication for this climate-sensitive area, where the glacier melting supplies water for billions of people downstream.

With vast mountain glaciers (ca. 10^5^ km^2^), the Himalayas and Tibetan Plateau (HTP) present a unique and sensitive area under the regime of climate change^1^,^2^. Meltwater from those glaciers supplies major Asian rivers, such as the Indus River, Ganges River, Yarlung Tsangpo (Brahmaputra), Yangtze River and Yellow River^3^,^4^. At the same time, serious air pollution (Atmospheric Brown Clouds, ABC) is widely spread on the Indo Gangetic Plain (IGP)^5^. To evaluate the influence of the brown cloud over IGP on the high Himalayas, several continuous observations have been implemented under the framework of the UNEP-ABC project^6^. Those research clearly demonstrated that the ABC could affect the high altitudes, and even be lifted to more than 10 km in height^7^. However, most current works were confined to the southern slope of the Himalayas and little is known about the northern slope, especially the spread and transport mechanism of these air pollutants^8^. Actually, if the pollutants could be transported across the Himalayas, the gentle surface of the Tibetan Plateau will favor their further spread to the north, where more glaciers locate^9^. The deposition and accumulation of carbonaceous particles on the snow/ice surface may dramatically decrease the snow albedo, thereby resulting in increased glacier melt^10^,^11^,^12^,^13^.

As major constituents of atmospheric aerosols, organic acids can originate either from the primary process such as biomass burning and traffic emission^14^ or the secondary formation by gas-to-particle conversion from various precursors^15^,^16^. Due to the hygroscopicity and the capability to act as cloud condensation nuclei (CCN), organic acids in aerosols are of great importance in the climate system^17^. Moreover, several specific compounds and their concentration ratios have been well recognized as unique fingerprints to identify the sources, transport and reaction processes of atmospheric aerosols. Given the importance of organic acids, they were widely studied in different locations such as urban^18^, continental background^19^,^20^, ocean^21^ and the Arctic^22^. However, such research is very scarce in the HTP. In ice core samples from Mt. Everest, Kang^23^ found the oxalate concentration level during the 1950s–1980s was three times the background value of the 19^th^ century values. This tripling was attributed to the anthropogenic production.

In this study, dicarboxylic acids and related compounds in aerosols were measured for the first time on Mt. Everest, a high‐altitude site on the northern slope of the Himalayas (28.36°N, 86.95°E; 4276 m above sea level), from August 2009 to July 2010. The major purpose of this research is to reveal the transport process of atmospheric pollutants across the Himalayas by exploiting the source-indicating faction of organic acids.

## Results

### Molecular distribution of dicarboxylic acids and related compounds

Table 1 shows the average concentration and ranges of various dicarboxylic and related compounds in aerosols from Mt. Everest. Annual average concentrations of dicarboxylic acids, oxoacids and α-dicarbonyls were 109, 7.39 and 0.85 ng m^−3^, accounting for 7.45%, 0.39% and 0.06% of total organic carbon (OC), respectively. The contribution of dicarboxylic acids and related compounds to aerosol OC could be up to 15.5%, indicating that organic acids are major components in the high mountain aerosols. The average concentration of dicarboxylic acids in Mt. Everest aerosol is two to four times higher than that (64 ng m^−3^) reported in Alert, Arctic^24^, and that (30 ng m^−3^) in Syowa, Antarctica^25^. The value found in this study is comparable with data from some remote marine sites, such as 139 ng m^−3^ at Chichi-jima Island in the western North Pacific^26^, but much lower than those observed in Asian cities. For example, high levels of dicarboxylic acids were reported in New Delhi (2330 ng m^−3^)^27^ and Chennai (612 ng m^−3^)^28^, India, and Hong Kong (692 ng m^−3^)^29^. Furthermore, in this study the average concentration of total dicarboxylic acids in the pre-monsoon period (235 ng m^−3^) is significantly higher than during other seasons (63.1 ng m^−3^ in monsoon, 58.4 ng m^−3^ in post-monsoon and 69.7 ng m^−3^ in winter, Table S1). The high loading of dicarboxylic acids in the pre-monsoon season in the Mt. Everest region was likely related to the source strength rather than photochemical oxidation. According to a previous study, a positive linear correlation exists between dicarboxylic acids and air temperature^30^. However, the maximum air temperature at Mt. Everest was observed in the summer monsoon season.

**Figure 1:**
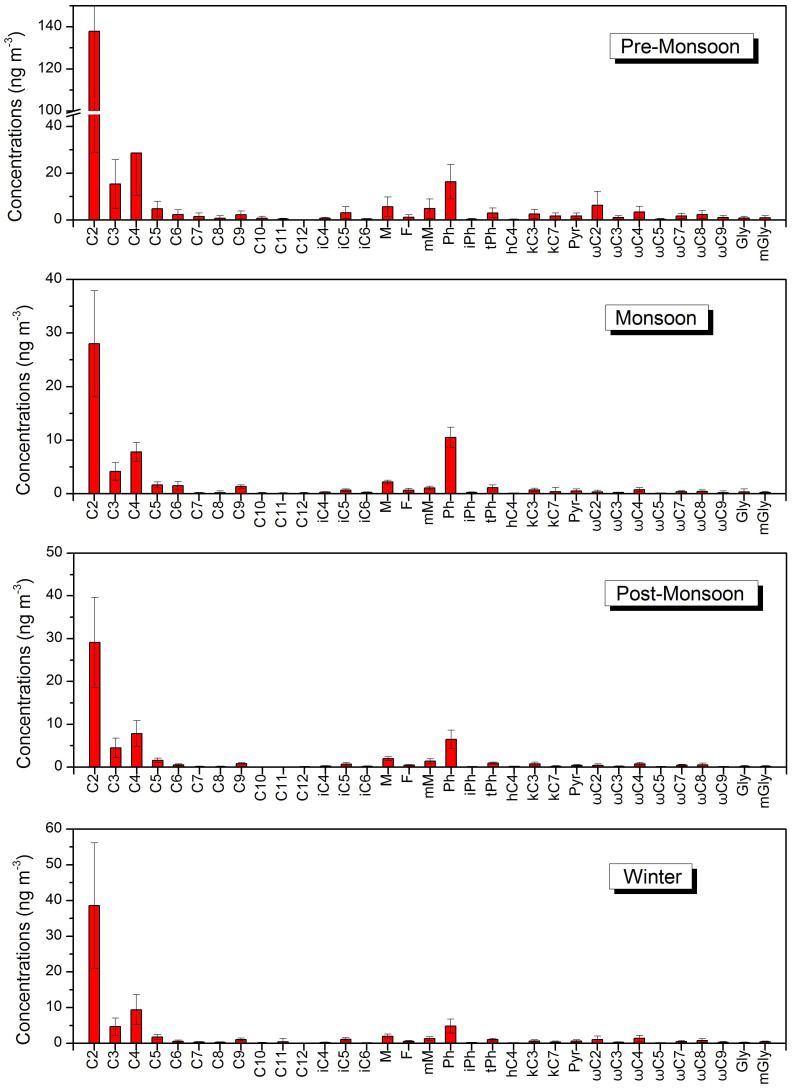
Molecular distributions of dicarboxylic acids and related compounds in different seasons at Mt. Everest (QOMS station), the northern slope of the Himalayas.

Seasonal average molecular distributions of dicarboxylic acids, oxoacids and α-dicarbonyls in the aerosols are presented in Figure 1. Although large differences occurred among seasons, oxalic acid (C_2_) was detected as the most abundant dicarboxylic acid species, followed by succinic (C_4_) and malonic acid (C_3_) in all seasons. Oxalic acid was commonly detected as the predominant species in various environments worldwide. However, a clear pattern that C_4_ being more abundant than C_3_, a typical signal of biomass burning emission^31^,^32^, was found in the Mt. Everest aerosols. Therefore, our finding indicates the importance of biomass burning influences in this area, the point to be further discussed below.

## Discussion

### Primary versus secondary contribution reflected by ratios of dicarboxylic acids

Because succinic acid (C_4_) tends to be degraded into malonic acid (C_3_), the ratio of C_3_/C_4_ has widely been used as an indicator to evaluate the photochemical production of dicarboxylic acids^18^,^21^. The C_3_/C_4_ ratios in the Mt. Everest aerosols ranged from 0.11 to 0.81 with an average of 0.51, which is similar to some urban sites (such as 0.6 in New Delhi)^27^, while much lower than those from continental and remote marine sites. For example, the C_3_/C_4_ ratio from Qinghai Lake, northern TP is 2.2 due to the photochemical production of C_3_ from C_4_^33^. Even higher ratios are found over the Atlantic Ocean (2.1–3.4)^21^ and the equatorial central Pacific (up to 10)^34^. Low C_3_/C_4_ ratios in this study show that the aerosols from Mt. Everest were relatively fresh, again indicating that the direct influence from primary emission source is more important than photochemical oxidation.

The unsaturated dicarboxylic acid, maleic acid (M, *cis* configuration), is formed by the degradation of aromatic hydrocarbons (e.g. toluene and benzene). Under the solar radiation, it could be further isomerized to its *trans* isomer, fumaric acid (F), through photochemical processes^30^. Therefore, the ratios of M/F can be applied to assess the aging of aerosols, i.e, lower M/F ratio means higher photochemical aging. In the present study, the M/F ratios (1.55–8.16, average 4.44) are much higher than those at marine sites (e.g. the North Pacific, 0.06–1.3, average 0.26)^34^. While the M/F ratios of Mt. Everest aerosols are similar to those reported at urban sites (New Delhi, 2.0–3.6; Beijing, 2.3)^27^,^35^ and sites intensively impacted by biomass burning (Mt. Tai, China, 2.0; Rondonia, Amazonia, 2.8)^31^,^32^. The high M/F ratios found in this study also suggest that isomerization of maleic acid to fumaric acid by photochemical transformation is not significant at Mt. Everest. This finding confirms that Mt. Everest aerosols are rather fresh, without being substantially aged.

### Source attribution – the influence of biomass burning

**Figure 2:**
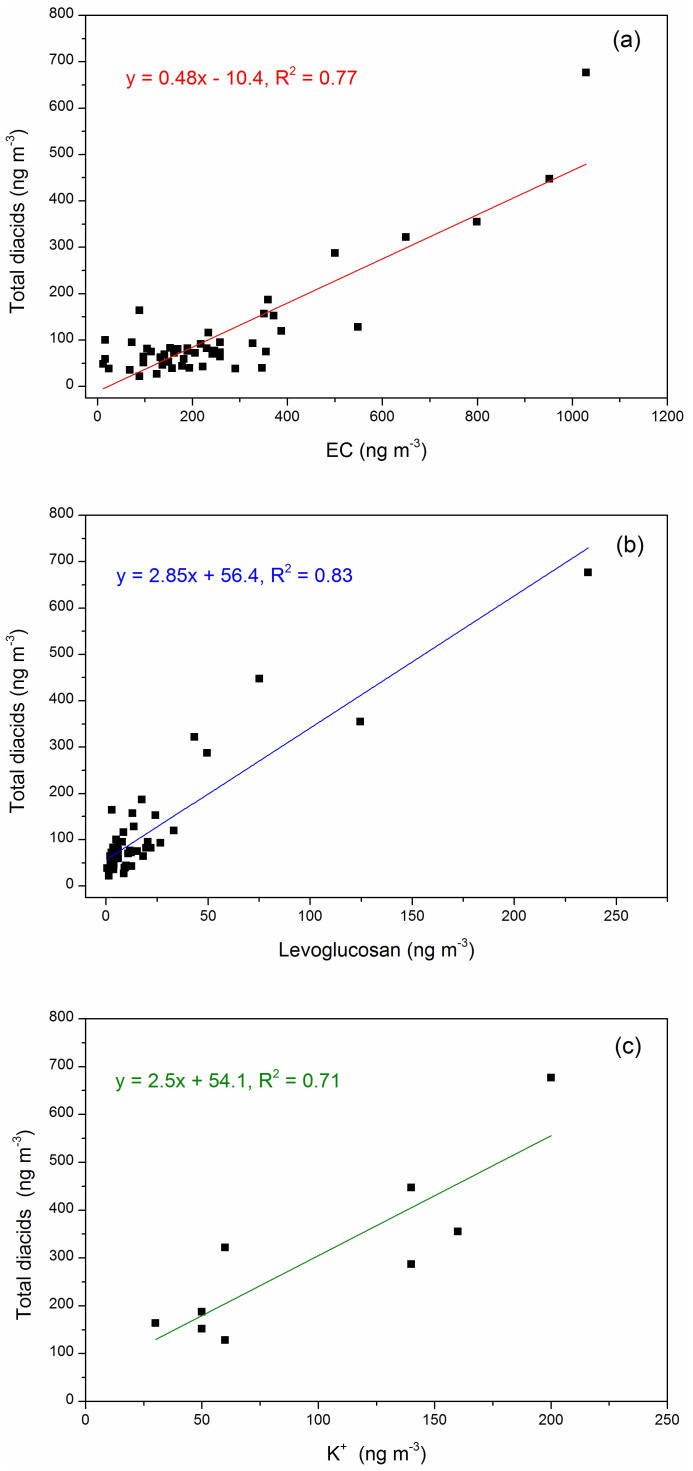
Relationship between the concentrations of total dicarboxylic acids and (a) Elemental Carbon (EC), (b) Levoglucosan and (c) water-soluble potassium (K^+^).

Dicarboxylic acids have various sources, including primary sources such as biomass burning, vehicular exhausts and cooking, and secondary sources i.e. atmospheric photooxidation of organic precursors. Since EC is only emitted by combustion sources (fossil fuel and/or biomass burning), it has been used frequently as a conservative tracer for primary combustion-generated OC. In this study, the dicarboxylic acids exhibit a strong correlation with EC (R^2^ = 0.77, Fig. 2a), which demonstrates that primary combustion sources are the predominant contributor to dicarboxylic acids, while secondary photochemical production is negligible. Levoglucosan is a specific tracer of biomass burning, because it can only be produced through the pyrolysis of cellulose during the combustion process^36^. For the aerosols from Mt. Everest, a strong positive correlation between levoglucosan and dicarboxylic acids was found (Fig. 2b), with a correlation coefficient (R^2^) of 0.83. Furthermore, dicarboxylic acids also closely correlated with another biomass burning tracer, water-soluble potassium (K^+^) (Fig. 2c) (Note here, only the samples with K^+^ concentration above the detection limits were used for the correlation analysis.). Therefore, these results suggested that among the various primary combustion sources, biomass burning rather than fossil fuel (coal combustion or vehicular exhaust) is the prevalent source of dicarboxylic acids at Mt. Everest. Especially in the pre-monsoon season, the higher ratios of levoglucosan to EC emphasize the importance of biomass burning influence (Fig. S1). Our findings differs from a previous study at Qinghai Lake (3200 m a.s.l.) over the northern TP (similar background conditions to this study), where dicarboxylic acids are found to be mainly derived from photochemical oxidation due to strong radiation^33^.

### Potential source region and transport mechanism

**Figure 3:**
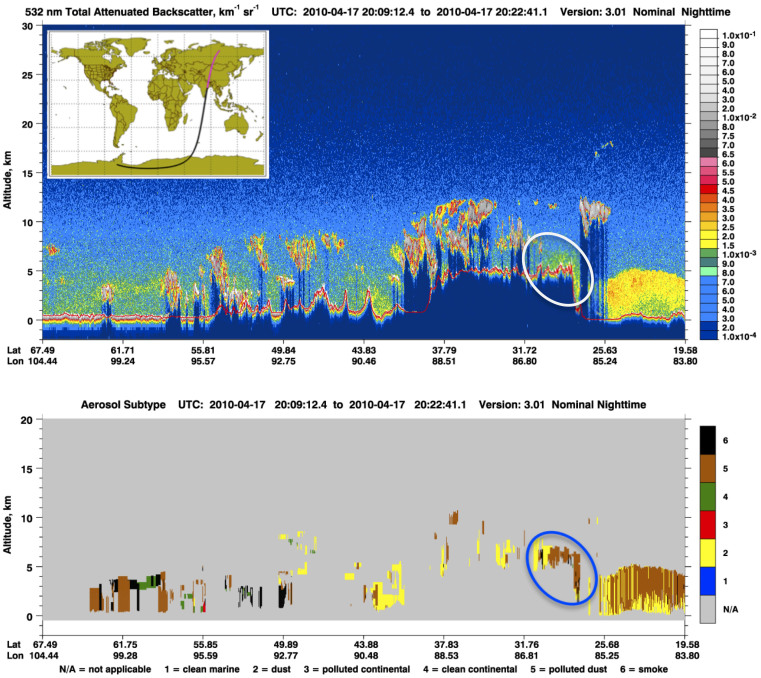
CALIPSO retrieved backscatter signal at 532 nm (upper panel) and aerosol sub-type information (lower panel) on 17 April 2010. The Himalayas and southern TP (marked with circles) are covered by a thick aerosol layer, suggesting that air pollutants could be uplifted to more than 6 km high in altitude. CALIPSO profiles were obtained from (http://www-calipso.larc.nasa.gov).

Because molecular distributions and ratios of organic acids in the Mt. Everest aerosols exhibited a strong influence from biomass burning, we further checked such emission strength for different seasons using the active fire spots from MODIS (FIRMS, https://earthdata.nasa.gov/firms). Results showed that in the pre-monsoon period there were a great number of agricultural burning and forest fires along the southern Himalayan foothills and Northern IGP (Figure S2). Similarly, a recent work (BC and O_3_) conducted at the Nepal Climate Observatory-Pyramid station (NCO-P) on the southern slope of the Himalayas also pointed out the importance of open fire emission from that area^37^. Our finding is also in agreement with the viewpoint of Vadrevu et al.^38^, that the pre-monsoon period (especially April) is the major fire season in the lowland of the southern Himalayas. The meteorological regime of this region is characterized by humid air masses from the Indian Ocean in the summer monsoon season and strong westerlies in other seasons (Fig. S3). Therefore, the wind system could facilitate the transport of air pollutants from South Asia to the Himalayas, regardless the shift of air circulation pattern among seasons. The aerosol vertical distribution achieved by Cloud-Aerosol Lidar with Orthogonal Polarization (CALIOP) retrievals demonstrates that smoke plume could reach beyond 6 km in altitude, which is higher than most of the mountain valleys in the Himalayas. An example of such pollution phenomenon can be observed on 17 April 2010 (Fig. 3), which clearly demonstrates that the Himalayas and the southern TP (marked with circles) are covered by a thick polluted aerosol layer, which apparently originated from South Asia.

**Figure 4:**
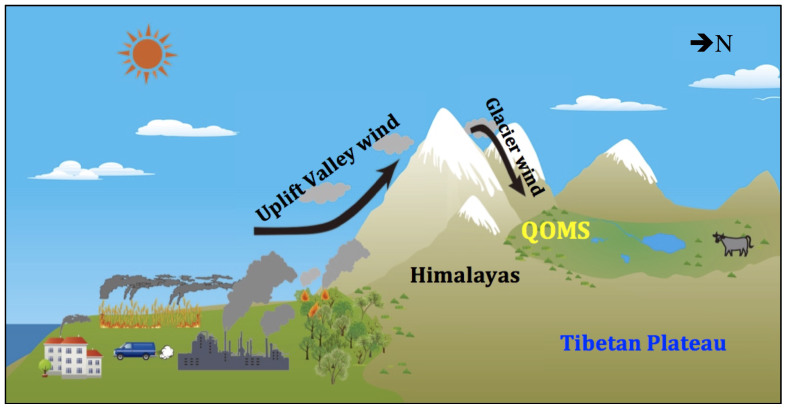
Illustration of the aerosol transport mechanism from the lowlands of South Asia to across the Himalayas by the mountain/valley wind system (generated by Z.Y. Cong).

In addition to large-scale atmospheric circulation, the local orography may also play an important role in air pollutant transport. The mountain/valley wind system in the southern Himalayas is characterized by an evident up-valley wind in daytime with a maximum in the afternoon, which delivers substantial pollutants from South Asian lowland to higher altitude (e.g. NCO-P)^6^,^39^. In contrast, a predominant down-valley wind occurs on the northern slope of the Himalayas with peaks in the afternoon, because the downward “glacier wind” produced by the vast snow/ice cover in the northern Himalayas can overcome the normal up-valley air flow in daytime^40^,^41^. Therefore, acting as efficient channels of south-to-north air flow, the mountain valleys could allow the air pollutants to easily penetrate throughout the Himalayas (Fig. 4). A previous work^42^ has revealed a trans-Himalayan pollution episode from Khumbu Valley, Nepal to the Tibetan Plateau (Rongbuk Valley) based on the simultaneous observation of condensation nucleus. When carbonaceous aerosols emitted from South Asia are transported to the far north (i.e. inland on the TP) and eventually deposited and accumulated on glacier surfaces, undoubtedly, they will change the energy balance of glaciers^43^.

## Method

### Research site and sampling

During August 2009 to July 2010, total suspended particle (TSP) samples (n = 50) were collected weekly using a medium-volume sampler with pre-combusted quartz filters at Mt. Everest (Qomolangma Station for Atmospheric and Environmental Observation and Research, 28.36°N, 86.95°E; 4276 m above sea level). Given the remote location and very sparse local population, QOMS is an ideal place to monitor the atmospheric environment in the Himalayas^44^. According to meteorological measurements, the seasonality at QOMS was divided into pre-monsoon, monsoon, post-monsoon and winter (Table S2).

After the water extraction and butyl ester derivatization, dicarboxylic acids (C_2_-C_12_), oxocarboxylic acids (C_2_-C_9_) and α-dicarbonyls (glyoxal and methylglyoxal) (Fig. S4) in the aerosol samples were determined using gas chromatography with a flame ionization detector (GC-FID) following the modified analytical methods established by Kawamura and Ikushima^18^ and Kawamura^45^.

The concentration of OC and EC was measured using an OC/EC analyzer following the IMPROVE-A protocol. Levoglucosan was determined by GC/MS after the extraction of the samples with a methanol/methylene chloride mixture followed by BSTFA derivatization^46^. The water-soluble ionic species were determined using an ion chromatograph (761 Compact IC, Metrohm). Details about the analytical procedures for dicarboxylic acids, oxocarboxylic acids, α-dicarbonyls, OC, EC, and K^+^, as well as quality assurance and control are presented in the Supplementary Materials. All the concentrations of dicarboxylic acids and related compounds, carbonaceous and ionic components in this study are field-blank corrected.

## Supplementary Material

Supplementary Information Supplementary Information

## Figures and Tables

**Table 1:** Concentrations of dicarboxylic acids, oxocarboxylic acids and α-dicarbonyls (ng m^−3^) in the aerosols from QOMS station, the north slope of the Himalayas

	Concentrations (ng m^−3^)			
	Mean	S.D.	Min	Max
**Dicarboxylic acids**				
**Oxalic, C_2_**	59.8	73.4	7.64	426
**Malonic, C_3_**	7.30	7.37	0.76	39.7
**Succinic, C_4_**	13.7	13.13	2.55	72.1
**Glutaric, C_5_**	2.49	2.22	0.50	12.7
**Adipic, C_6_**	1.23	1.34	0.15	7.43
**Pimelic, C_7_**	0.54	0.98	0.03	5.35
**Sebacic, C_8_**	0.35	0.61	0.00	2.91
**Azelaic, C_9_**	1.38	1.01	0.42	6.75
**Decanedioic, C_10_**	0.28	0.50	0.00	2.78
**Undecanedioic, C_11_**	0.26	0.53	0.00	3.55
**Dodecanedioc, C_12_**	0.11	0.10	0.00	0.51
**Methylmalonic, iC_4_**	0.36	0.33	0.00	1.97
**Methylsuccinic, iC_5_**	1.43	1.66	0.20	10.0
**2-Methylglutaric, iC_6_**	0.24	0.16	0.05	0.85
**Maleic, M**	2.98	2.71	0.78	16.4
**Fumaric, F**	0.72	0.62	0.22	3.34
**Methylmaleic, mM**	2.20	2.68	0.56	14.2
**Phthalic, Ph**	9.47	6.14	2.27	33.2
**Isophthalic, iPh**	0.23	0.17	0.06	0.87
**Terephthalic, tPh**	1.55	1.43	0.32	8.07
**Malic, hC_4_**	0.15	0.10	0.00	0.52
**Oxomalonic, kC_3_**	1.18	1.31	0.16	6.52
**4-Oxopimelic, kC_7_**	0.67	1.00	0.00	3.99
**Subtotal**	109	117	16.7	677
**Contribution to OC (%)**	7.45	2.52	1.71	14.7

**Oxocarboxylic acids**				
**Pyruvic, Pyr**	0.83	0.87	0.04	4.33
**Glyoxylic (2-oxoethanoic), *ω*C_2_**	2.09	3.97	0.00	19.6
**3-Oxopropanoic, *ω*C_3_**	0.44	0.54	0.00	2.46
**4-Oxobutanoic, *ω*C_4_**	1.62	1.76	0.16	9.87
**5-Oxopentanoic, *ω*C_5_**	0.17	0.20	0.02	1.21
**7-Oxoheptanoic, *ω*C_7_**	0.77	0.86	0.00	4.49
**8-Oxooctanoic, *ω*C_8_**	1.04	1.23	0.00	5.72
**9-Oxononanoic, *ω*C_9_**	0.43	0.60	0.00	3.28
**Subtotal**	7.39	10.0	0.22	50.9
**Contribution to OC (%)**	0.39	0.20	0.03	0.79

**α-Dicarbonyls**				
**Glyoxal, Gly**	0.40	0.56	0.03	2.68
**Methylglyoxal, mGly**	0.45	0.56	0.00	2.45
**Subtotal**	0.85	1.12	0.03	5.12
**Contribution to OC (%)**	0.06	0.05	0.01	0.29
