# Supplementary material for: Penetration of biomass-burning emissions from South Asia through the Himalayas: new insights from atmospheric organic acids
Source: Sci Rep. 2015 Apr 9;5:9580. doi: 10.1038/srep09580 (PMC5381702; doi:10.1038/srep09580)
Supplement: Supplementary Information [file srep09580-s1.pdf]

Supplementary Materials for the manuscript:

Penetration of biomass-burning emissions from South Asia through the Himalayas: new insights from atmospheric organic acids

Zhiyuan Cong[1, 2, 3], Kimitaka Kawamura[2*], Shichang Kang[4, 3*], Pingqing Fu[5]

[1]Key Laboratory of Tibetan Environment Changes and Land Surface Processes, Institute of Tibetan Plateau Research, Chinese Academy of Sciences, Beijing 100101，China

[2]Institute of Low Temperature Science, Hokkaido University, Sapporo, 060-0819, Japan

[3]CAS Center for Excellence in Tibetan Plateau Earth Sciences, Beijing 100101, China

[4]State Key Laboratory of Cryospheric Sciences, Cold and Arid Regions Environmental and Engineering Research Institute, CAS, Lanzhou 730000, China

[5]LAPC, Institute of Atmospheric Physics, CAS, Beijing 100029, China

*Correspondence and requests for materials should be addressed to

K.K. (kawamura@lowtem.hokudai.ac.jp) or S.C.K. (shichang.kang@lzb.ac.cn)

Aerosol sampling

From August 2009 to July 2010, total suspended aerosol particle (TSP) samples were collected weekly at QOMS using medium-volume samplers (KC-120H, Laoshan Co., flow rate: 100 L min^{-1} at standard condition). The sampling duration of each sample was 24 hours. Aerosols were collected using 90-mm diameter quartz filters (QM/A, Whatman, UK), which were pre-combusted at 450 °C for six hours. Field blanks were collected every month by placing filters into the filter holder for a few minutes with no air flowing. After sampling, the filters were wrapped with aluminum foil and frozen until analysis. Eventually, 50 samples were successfully obtained.

Dicarboxylic acid analysis

The quartz filters were cut in pieces and extracted by Milli-Q water under ultrasonication three times. The extracts were combined into a 50 ml pear shape flask after filtration with quartz wool, and then concentrated to almost dryness using a rotary evaporator under vacuum. Before the concentration, pH of the extracts was adjusted to 8.5-9.0 using 0.05 M KOH. Next, 14% BF_3/n-butanol was added and heated for 1 h for derivation, i.e. converting the carboxyl groups to butyl esters and aldehyde groups to dibutoxy acetals. The derived butyl esters and acetals were extracted with n-hexane. The butyl esters and acetals were determined by a capillary gas chromatograph (Agilent 6980) with a flame ionization detector. Each compound was identified based on retention times of GC peaks with those of authentic standards and mass spectra obtained by GC-MS (Thermo Trace MS). Recoveries of authentic standards spiked on a pre-combusted quartz filter were 75% for oxalic and malonic acids, and greater than 90% for succinic and adipic acids. The levels of field blanks were generally less than 10%.

OC and EC analysis

The quartz filters were analyzed for OC and EC using a carbon analyzer (DRI model 2001). Briefly, a filter aliquot (0.5 cm^2) was analyzed for eight carbon fractions following the IMPROVE-A thermal/optical reflectance (TOR) protocol. Four OC

fractions (OC1, OC2, OC3 and OC4) were determined at 140, 280, 480 and 580 °C in pure He atmosphere, which was subsequently switched to 2% O_2/98% He atmosphere to determine EC1, EC2 and EC3 at 580, 740 and 840 °C, respectively. The residence time of each heating step was defined by the flattening of the carbon signal. The pyrolyzed carbon fraction (OPC) is determined when reflected laser light returns to its initial value after oxygen is introduced. In general, OC is defined as OC1 + OC2 + OC3 + OC4 + OPC and EC is defined as EC1 + EC2 + EC3 - OPC. The detection limit for the carbon analyzer was 0.05 μg C cm^{-2} for OC and 0.05 μg C cm^{-2} for EC.

Water-soluble ions

An aliquot of filter (2.54 cm^2) was extracted with 10 ml ultrapure water with sonication for 30 minutes. The extracted solutions were filtrated with syringe-driven filters (Millex - GV PVDF, 0.22 μm; Millipore, Ireland) to remove the quartz fiber debris and other insoluble impurities. Then the water-soluble ionic species (Cl^-, SO_4^{2-}, NO_3^-, Ca^{2+}, Na^+, K^+, Mg^{2+} and NH_4^+) were analyzed using an ion chromatograph (761 Compact IC, Metrohm). Anions were measured with a suppressor on a Shodex SI-90 4E column using an eluent mixture of 1.8 mM Na_2CO_3, 1.7 mM $NaHCO_3$ and 40 mM H_2SO_4 at a flow rate of 1.2 mL min^{-1}. Cations were determined on a Metrohm C2-150 column with tartaric acid (4 mM) and dipicolinic acid (1 mM) as an eluent. The overall uncertainty in determining ionic species is less than 4%. The detection limit for cations and anions was 0.01 μg m^{-3}, which was calculated according to the air volume of actual samples.

Meteorology

At the QOMS station, various meteorological parameters were recorded by a 40 m atmospheric boundary layer tower that measures wind speed, wind direction (014A-L, Met One), relative humidity, air temperature, air pressure (HMP45C, Vaisala) and rain intensity (TE525MM-L, Young). Monthly mean air temperature reaches a maximum of 12.3°C in July, with a minimum in January of -3.2°C. Humidity is highest in August while lowest in December. Precipitation was unevenly distributed throughout the year, with more than 90% of annual precipitation occurring from June

to September. According to the meteorological parameters at QOMS, the climatology is roughly divided into four seasons, i.e. pre-monsoon, monsoon, post-monsoon and winter (The definition of the different seasons is shown in Table S2). In general, this region is controlled by the Indian Monsoon system in summer (June-August), characterized by relatively high temperature and humid weather with prevailing southerly winds. In the remaining period, westerlies dominate the large-scale atmospheric circulation patterns with limited precipitation.

Table S1 Seasonal mean concentrations of dicarboxylic acids, oxocarboxylic acids and α-dicarbonyls (ng m^{-3}) as well as concentrations of OC, EC, K^{+} (μg m^{-3}) and Levoglucosan (ng m^{-3}) in the aerosols from Mt. Everest, the northern slope of the Himalayas.

	Pre-Monsoon		Monsoon		Post-Monsoon		Winter	
	Mean	SD	Mean	SD	Mean	SD	Mean	SD
Dicarboxylic acids								
Oxalic, C_2	138	109.07	28.0	9.90	29.1	10.5	38.6	17.6
Malonic, C_3	15.4	10.3	4.15	1.63	4.49	2.30	4.66	2.43
Succinic, C_4	28.6	18.1	7.79	1.78	7.84	3.00	9.42	4.25
Glutaric, C_5	4.86	3.20	1.65	0.56	1.58	0.54	1.73	0.70
Adipic, C_6	2.34	2.01	1.50	0.80	0.54	0.19	0.57	0.33
Pimelic, C_7	1.46	1.58	0.15	0.08	0.13	0.10	0.34	0.17
Sebacic, C_8	0.79	1.03	0.20	0.30	0.12	0.10	0.25	0.15
Azelaic, C_9	2.24	1.58	1.35	0.30	0.83	0.30	1.08	0.39
Decanedioic, C_{10}	0.73	0.82	0.11	0.14	0.06	0.04	0.17	0.08
Undecanedioic, C_{11}	0.40	0.35	0.09	0.09	0.07	0.07	0.44	0.88
Dodecanedioc, C_{12}	0.15	0.11	0.08	0.14	0.09	0.05	0.10	0.04
Methylmalonic, iC_4	0.71	0.46	0.30	0.07	0.21	0.14	0.21	0.12
Methylsuccinic, iC_5	3.09	2.52	0.63	0.26	0.72	0.33	1.10	0.46
2-Methylglutaric, iC_6	0.41	0.20	0.24	0.10	0.18	0.09	0.13	0.06
Maleic, M	5.71	4.17	2.19	0.35	1.97	0.48	1.94	0.64
Fumaric, F	1.20	0.99	0.64	0.35	0.44	0.12	0.56	0.18
Methylmaleic, mM	4.89	4.13	1.06	0.34	1.38	0.56	1.30	0.54
Phthalic, Ph	16.3	7.33	10.5	1.90	6.50	2.15	4.81	1.97
Isophthalic, iPh	0.36	0.27	0.23	0.11	0.13	0.05	0.21	0.05
Terephthalic, tPh	3.04	2.09	1.14	0.52	0.93	0.25	1.02	0.39
Malic, hC_4	0.24	0.14	0.10	0.06	0.15	0.06	0.11	0.05

Oxomalonic, kC_3	2.58	1.89	0.68	0.32	0.74	0.43	0.65	0.30
4-Oxopimelic, kC_7	1.70	1.34	0.38	0.80	0.20	0.12	0.34	0.31
Subtotal	235	174	63.1	14.9	58.4	22.0	69.7	32.1
Oxocarboxylic acids								
Pyruvic, Pyr	1.73	1.24	0.49	0.40	0.40	0.16	0.62	0.32
Glyoxylic (2-oxoethanoic), ωC_2	6.27	5.98	0.35	0.30	0.40	0.34	1.04	0.97
3-Oxopropanoic, ωC_3	1.05	0.76	0.21	0.09	0.17	0.12	0.27	0.15
4-Oxobutanoic, ωC_4	3.37	2.54	0.75	0.36	0.78	0.33	1.39	0.84
5-Oxopentanoic, ωC_5	0.34	0.32	0.10	0.05	0.10	0.03	0.14	0.08
7-Oxoheptanoic, ωC_7	1.73	1.19	0.36	0.20	0.44	0.23	0.49	0.26
8-Oxooctanoic, ωC_8	2.32	1.74	0.40	0.32	0.54	0.40	0.77	0.49
9-Oxononanoic, ωC_9	1.09	0.83	0.21	0.29	0.10	0.07	0.29	0.19
Subtotal	17.9	14.6	2.87	2.02	2.93	1.68	5.01	3.31
α-Dicarbonyls								
Glyoxal, Gly	0.84	0.78	0.31	0.57	0.20	0.22	0.23	0.13
Methylglyoxal, mGly	0.98	0.82	0.21	0.22	0.18	0.19	0.39	0.23
Subtotal	1.82	1.60	0.52	0.80	0.38	0.41	0.62	0.36
Other parameters								
OC	2.61	1.58	0.81	0.14	1.06	0.53	1.14	0.50
EC	0.44	0.31	0.10	0.06	0.19	0.07	0.26	0.12
K^+	0.06	0.07	BDL		BDL		0.00	0.02
Levoglucosan	47.2	64.2	4.00	2.77	7.27	5.38	14.6	8.43

BDL, Below Detection Limits (0.01 μg m^{-3}) for K^+

Table S2. The meteorological parameters of different seasons at Mt. Everest during the sampling period (Aug. 2009 – July 2010).

Season	Start Date	End Date	Air Pressure	R．H.	Temp.	Wind Speed
	(YYYY-MM-DD)		(hPa)	(%)	(℃)	(m/s)
Monsoon	2009/8/1<br>2010/6/2	2009/8/25<br>2010/8/25	606.25	55.26	12.39	6.51
Post-Monsoon	2009/8/26	2009/11/28	607.17	46.00	5.47	7.03
Winter	2009/11/29	2010/2/24	603.62	26.91	-3.93	8.19
Pre-monsoon	2010/2/25	2010/6/1	605.46	34.61	3.69	7.86

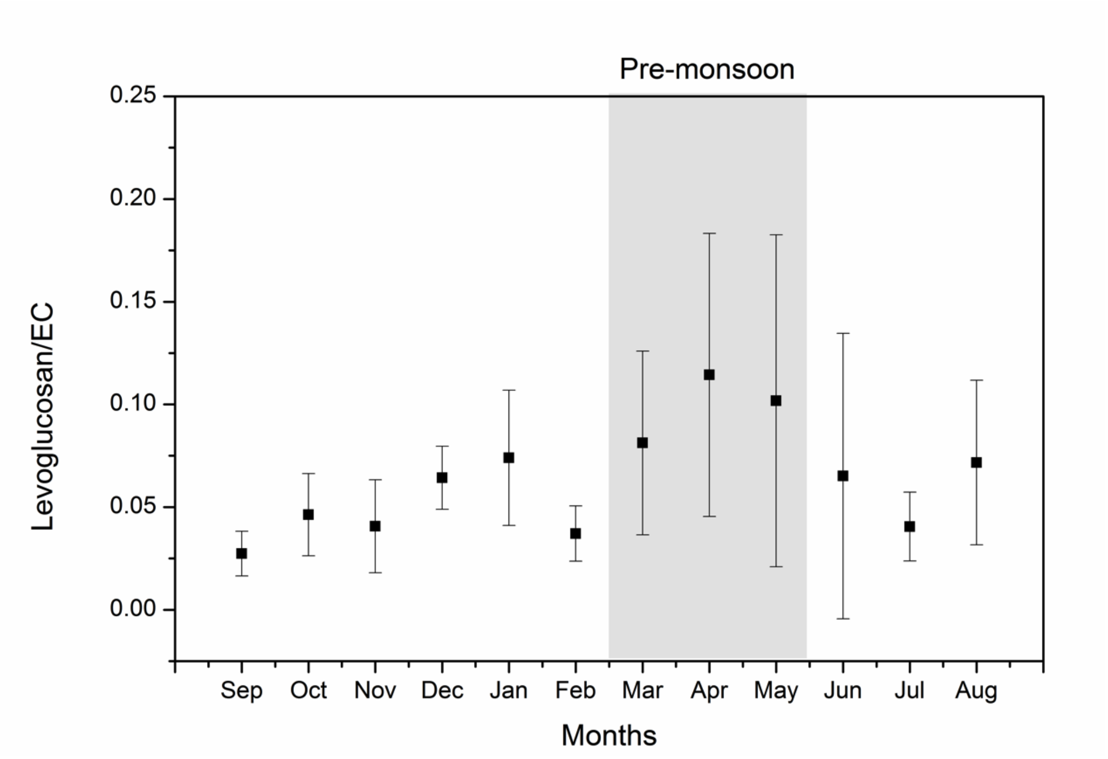

Fig. S1 Temporal variation of levolucosan/EC ratio in aerosols at Mt. Everest (QOMS station), the northern slope of Himalayas.

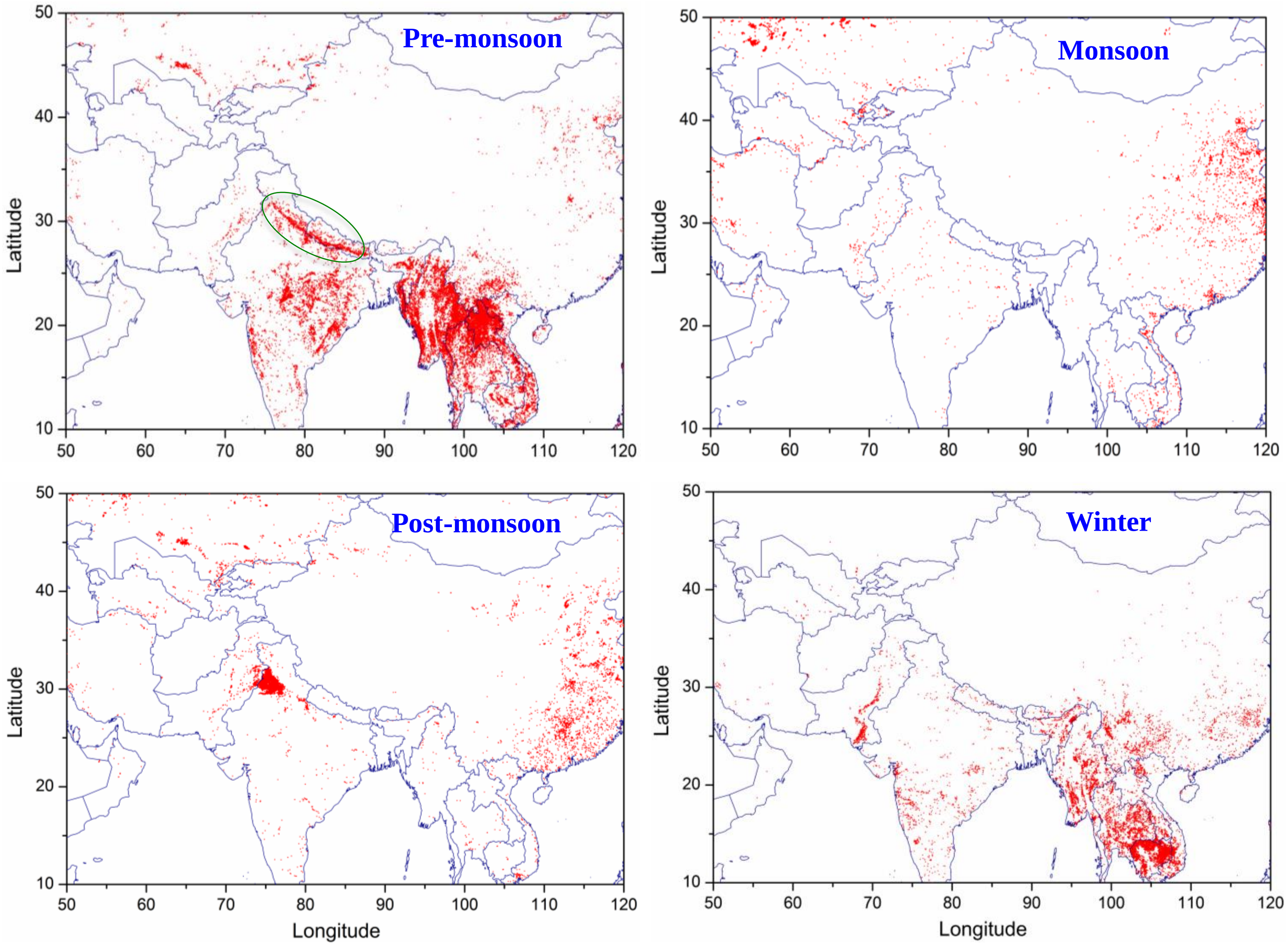

Fig. S2 The spatial distribution of fire spots observed by MODIS in different seasons. The fire spot data was obtained from EOSDIS, NASA (https://earthdata.nasa.gov/data/near-real-time-data/firms).

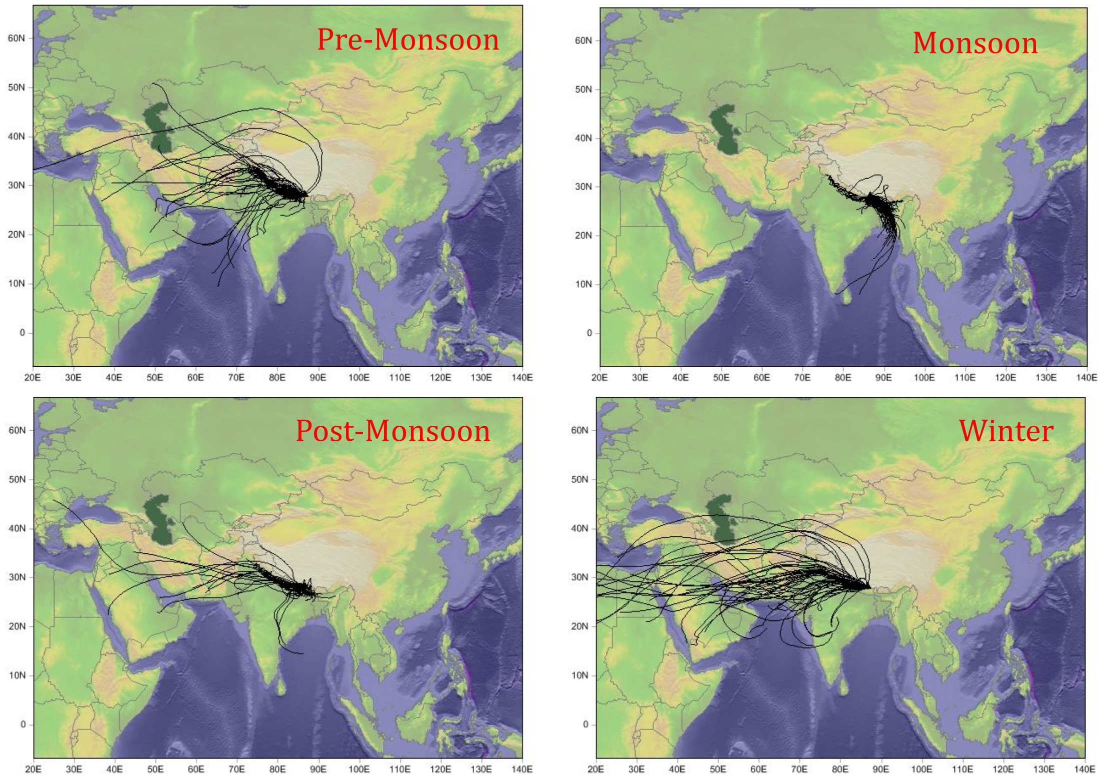

Fig. S3 Seven-day backward air mass trajectories calculated for the different seasons (Aug. 2009 – July 2010). The trajectories reveal two different types of air masses, corresponding to the summer monsoon and westerly system over the Himalayas and the Tibetan Plateau. In the monsoon season (JJA), slow-moving air masses come from Bangladesh and northeast India. In other seasons, most of the air masses come from the west at a rapid speed. Air mass trajectories were produced using HYSPLIT transport and dispersion model from the NOAA Air Resource Laboratory (http://ready.arl.noaa.gov/HYSPLIT.php).

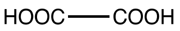

Oxalic acid (C_2) $C_2H_2O_4$

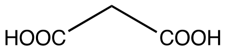

Malonic acid (C_3) $C_3H_4O_4$

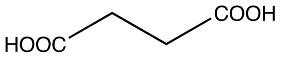

Succinic acid (C_4) $C_4H_6O_4$

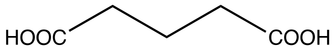

Glutaric acid (C_5) $C_5H_8O_4$

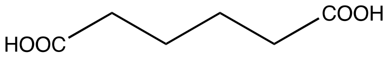

Adipic acid (C_6) $C_6H_{10}O_4$

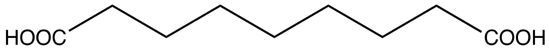

Azelaic acid (C_9) $C_9H_{16}O_4$

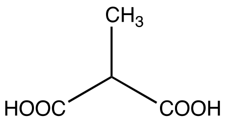

Methylmalonic acid (iC_4) $C_4H_6O_4$

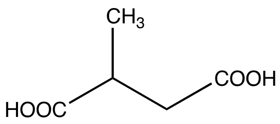

Methylsuccinic acid (iC_5) $C_5H_8O_4$

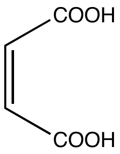

Maleic acid (M) $C_4H_4O_4$

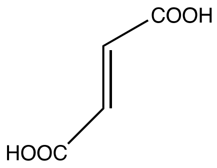

Fumaric acid (F) $C_4H_4O_4$

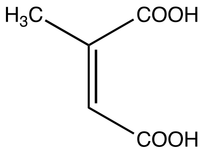

Methylmaleic acid (mM) $C_5H_6O_4$

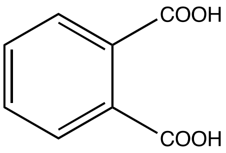

Phthalic acid (Ph) $C_8H_6O_4$

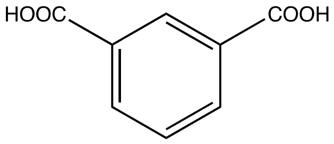

Iso-Phthalic acid (iPh) $C_8H_6O_4$

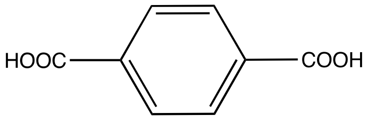

Tere-Phthalic acid (tPh) $C_8H_6O_4$

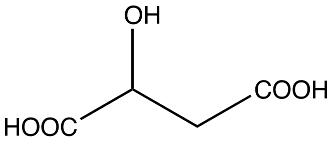

Malic acid (hC_4) $C_4H_6O_5$

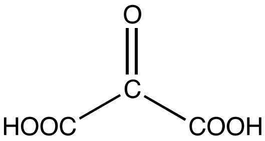

Ketomalonic acid (kC_3) $C_3H_2O_5$

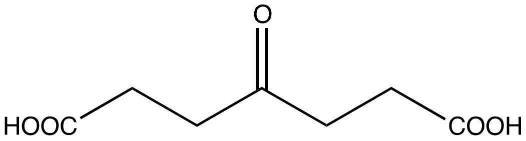

4-Ketopimelic acid (kC_7) $C_7H_{10}O_5$

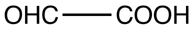

Glyoxylic acid (ωC_2) $C_2H_2O_3$

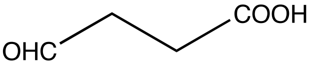

4-Oxobutanoic acid (ωC_4) $C_4H_6O_3$

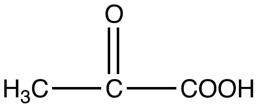

Pyruvic acid (Pyr) $C_3H_4O_3$

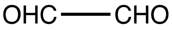

Glyoxal (Gly) $C_2H_2O_2$

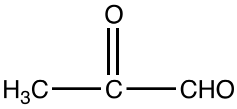

Methylglyoxal (MeGly) $C_3H_4O_2$

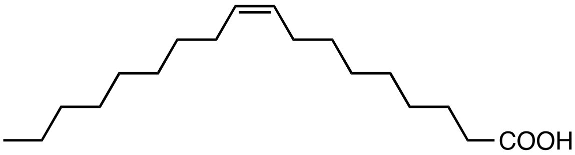

Oleic acid ($C_{18:1}$ fatty acid) $C_{18}H_{34}O_2$

Fig. S4 Chemical structure of selected organic acids detected in the Mt. Everest aerosols.
